# The complete chloroplast genome sequence of endangered plant *Trachycarpus nanus* (Arecaceae)

**DOI:** 10.1080/23802359.2021.1932625

**Published:** 2021-05-27

**Authors:** Xiong-Li Zhou, Ping Li, Liu Yang, Bo Long, Yue-Hua Wang, Shi-Kang Shen

**Affiliations:** a School of Ecology and Environmental Sciences Yunnan University, Kunming, Yunnan, China; b School of Life Sciences, Yunnan University, Kunming, Yunnan, China; c Yunnan Key Laboratory of Plant Reproductive Adaptation and Evolutionary Ecology, Yunnan University, Kunming, Yunnan, China

**Keywords:** *Trachycarpus nanus*, endemic, landscape plant, phylogenetic, mountain

## Abstract

*Trachycarpus nanus* is an endangered plant that is endemic to southwest of China. In the present study, the complete chloroplast genome of this species was assembled and characterized using whole genome next-generation sequencing. The complete chloroplast genome showed a circular genome of 158,713 bp size with 36.6% GC content. The genome is of typical structure and contain a pair of inverted repeat (IR) regions with 27,240 bp, separated by one large single-copy (LSC) with 86,395 bp, and one small single-copy (SSC) regions with 17,838 bp. The genome contained 132 genes, including 86 protein-coding genes, 8 rRNA genes and 38 tRNA genes. A phylogenetic tree reconstructed based on 21 chloroplast genomes reveals that *Trachycarpus nanus* is most related with *Chamaerops humilis.* The information provides important genetic basis for the species’ future studies on phylogenetic and utilization.

*Trachycarpus nanus* Beccari Webbia, which is endemic to southwestern China, is an endangered species in the family Arecaceae. This species is a dioecious perennial shrub without solitary, short and subterranean stem. This species is narrowly distributed in dry forests or open areas on mountains with altitude of 1800–2300 m in Yunnan province of China (Wu and Raven 2013). Thus, *Trachycarpus nanus* has been ranked as the second most endangered species in China and is subject to national protection. Based on the species categories of the International Union for Conservation of Nature and Natural Resources (IUCN), *Trachycarpus nanus* has been listed as an endangered plant (IUCN 2019; http://www.iucnredlist.org). Previous studies on *Trachycarpus nanus* mainly focused on its field survey and seed propagations (Dong et al. 2002), and genomic information is still scarce (Barrett et al. 2016). Herein, we firstly assembled and characterized the complete chloroplast genome for *Trachycarpus nanus* using next-generation sequencing technology. Such information will provide the genetic information for future studies on phylogenetic, evaluation and utilization of this species.

Fresh leaves of *Trachycarpus nanus* were collected from Xiangyun county of Dali in Yunnan Province, China (E: 100°47′14.28″, N: 25°37′3.576″, 2184 m). The specimen is stored at Yunnan University Herbarium (YNUWYH008-01, Yue-hua Wang wangyh58212@126.com). Total genomic DNA was extracted using a modified cetyltrimethylammonium bromide (CTAB) method (Doyle 1987). The sequencing library was constructed and quantified, and then the paired-end (PE) libraries were generated using Illumina HiSeq 2500 platform. The whole genome sequencing was conducted by Softgene (Beijing, China). We assembled the short reads into contigs using SPAdes (Bankevich et al. 2012), connected all contigs with Bandage (Wick et al. 2015), and manually removed redundant contigs. We mapped reads by bwa (Li and Durbin 2009) to the genome to check, proofread, and patch and finally obtained cycle complete plastomes. The cp genome was annotated through DOGMA (Wyman et al. 2004), and the boundaries of start and stop codons, and intron/exon were checked manually using Geneious version 8.1.4. We confirmed all tRNA genes using online tRNAscan-SE (Schattner et al. 2005). The final complete plastomes were deposited in GenBank with accession number MN935457.

The cp genome of *Trachycarpus nanus* is a circular molecule of 158,713 base pairs (bp), with a pair of Inverted Repeats (IR) of 27,240 bp, separated by a large single-copy (LSC, 86,395 bp) and a small single-copy (SSC, 17,838 bp). The overall GC content of *Trachycarpus nanus* cp genome is 36.6% and the corresponding values in LSC, SSC, and IR regions are 35.3%, 30.6%, and 42.4%, respectively. The cp genomes were annotated with 132 genes, including 86 protein-coding genes, 38 tRNA genes, and 8 rRNA genes. A total of 103 simple sequence repeats (SSRs) were detected using the online software MISA (http://pgrc.ipk-gatersleben.de/misa/) (Beier et al. 2017). The numbers of mono- (at least 10 repeats), di- (at least 5 repeats), tri- (at least 4 repeats), tetra- (at least 3 repeats), penta- (at least 3 repeats) and hexa-nucleotides (at least 3 repeats) SSRs are 70, 17, 5, 7, 3 and 1, respectively.

To reveal the systematic position of *Trachycarpus nanus*, we performed a phylogenomic analysis using the chloroplast genomes sequences of 21 species (*Baxteria australis* and *Kingia australis* as outgroup) in PAUP version 4.0a with 1000 bootstrap replicates (Swofford, 2002). The phylogenetic tree indicated that *Trachycarpus nanus* has closer relationship with *Chamaerops humilis* than other species with 100% bootstrap value (Figure 1). This study will provide valuable genomic resources for revealing the species’ phylogeny, exploring genetic variations, and designing utilization strategy.

**Figure 1.**
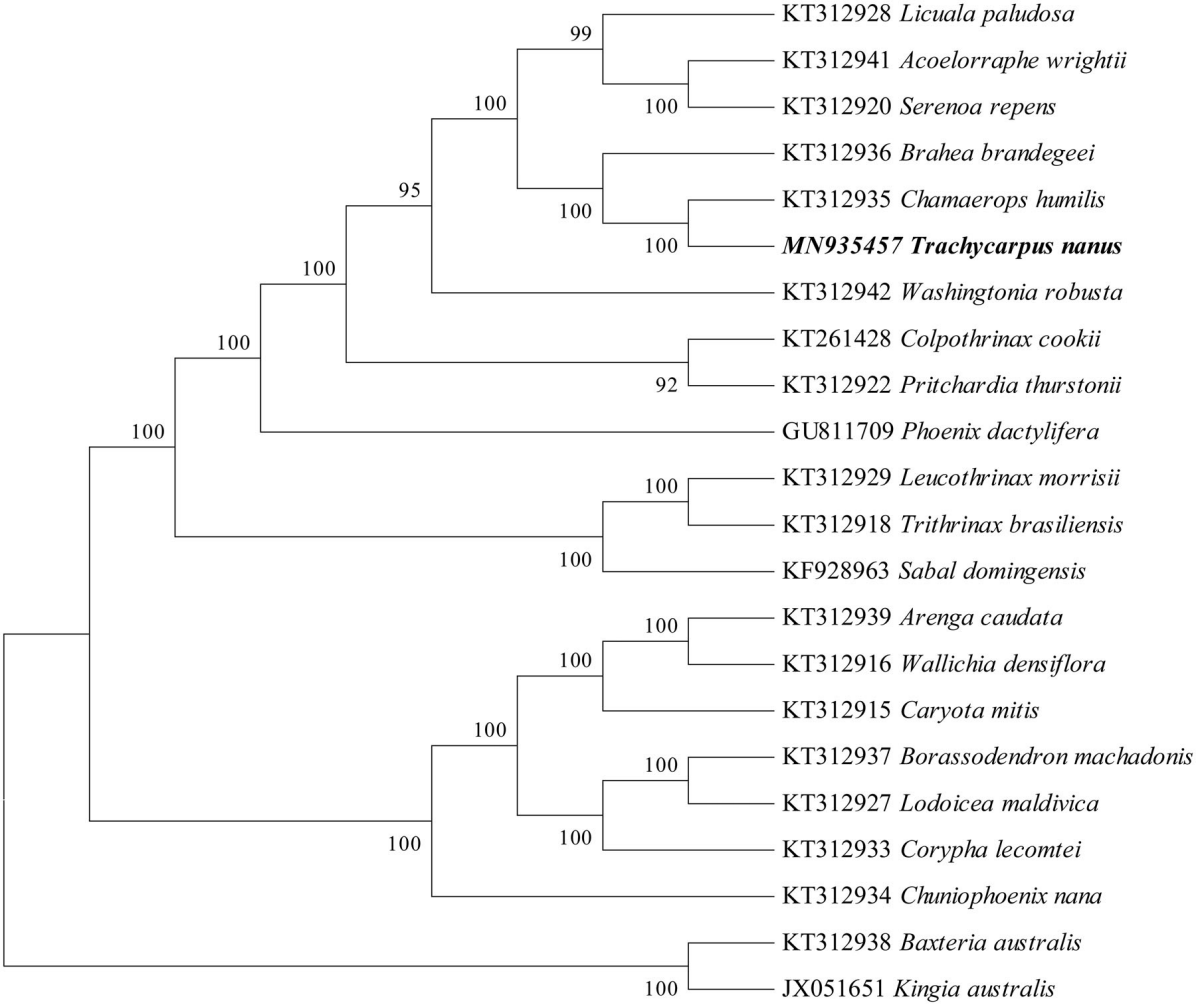
Phylogenetic position of *Trachycarpus nanus* based on the complete chloroplast genome sequences of 21 species. Bootstraps were shown next to the node.

## Data Availability

The data that support the findings of this study are openly available in GenBank of NCBI at https://www.ncbi.nlm.nih.gov, reference number MN935457.

## References

[CIT0001] Bankevich A, Nurk S, Antipov D, Gurevich AA, Dvorkin M, Kulikov AS, Lesin VM, Nikolenko SI, Pham S, Prjibelski AD, Pyshkin AV, et al. 2012. SPAdes: a new genome assembly algorithm and its applications to single-cell sequencing. J Comput Biol. 19(5):455–477. 22506599 10.1089/cmb.2012.0021 PMC3342519

[CIT0002] Barrett CF, Baker WJ, Comer JR, Conran JG, Lahmeyer SC, Leebens-Mack JH, Li J, Lim GS, Mayfield-Jones DR, Perez L, Medina J, et al. 2016. Plastid genomes reveal support for deep phylogenetic relationships and extensive rate variation among palms and other commelinid monocots. New Phytol. 209(2):855–870. 26350789 10.1111/nph.13617

[CIT0003] Beier S, Thiel T, Münch T, Scholz U, Mascher M. 2017. MISA-web: a web server for microsatellite prediction. Bioinformatics. 33:2583–2585. 28398459 10.1093/bioinformatics/btx198 PMC5870701

[CIT0004] Dong XD, Li JH, Yang XX. 2002. Survey of Yunnan *Trachycarpus nanus* and its biological features. Ecol Sci. 21:338–341.

[CIT0005] Doyle JA. 1987. Rapid DNA isolation procedure for small quantities of fresh leaf tissue. Phytochem Bull. 19:11–15.

[CIT0006] Li H, Durbin R. 2009. Fast and accurate short read alignment with Burrows-Wheeler transform. Bioinformatics. 25:1754–1760. 19451168 10.1093/bioinformatics/btp324 PMC2705234

[CIT0007] Schattner P, Brooks A, Lowe TM. 2005. The trnascan-se, snoscan and snogps web servers for the detection of trnas and snornas. Nucleic Acids Res. 33:W686. 15980563 10.1093/nar/gki366 PMC1160127

[CIT0008] Swofford DL. 2002. PAUP_: phylogenetic analysis using parsimony (and other methods). Sunderland (MA): Sinauer Associates.

[CIT0009] Wick RR, Schultz MB, Zobel J, Holt KE. 2015. Bandage: interactive visualisation of *de novo* genome assemblies. Bioinformatics. 31(20):3350–3352. 26099265 10.1093/bioinformatics/btv383 PMC4595904

[CIT0010] Wu ZY, Raven PH. 2013. Flora of China. Beijing: Science Press. Vol. 23. p. 145–146.

[CIT0011] Wyman SK, Jansen RK, Boore JL. 2004. Automatic annotation of organellar genomes with dogma. Bioinformatics. 20:3252–3255. 15180927 10.1093/bioinformatics/bth352

